# Academic research productivity of post-graduate students at Makerere University College of Health Sciences, Uganda, from 1996 to 2010: a retrospective review

**DOI:** 10.1186/s12961-017-0194-8

**Published:** 2017-04-04

**Authors:** E. A. Obuku, J. N. Lavis, A. Kinengyere, D. K. Mafigiri, F. Sengooba, C. Karamagi, N. K. Sewankambo

**Affiliations:** 1 Clinical Epidemiology Unit, Department of Medicine, School of Medicine, College of Health Sciences, Makerere University, PO Box 7072, Kampala, Uganda; 2 McMaster Health Forum, Centre for Health Economics and Policy Analysis, Department of Clinical Epidemiology and Biostatistics and Department of Political Science, McMaster University, Hamilton, Canada; 3 Department of Health Policy and Planning, School of Public Health, College of Health Sciences, Makerere University, Kampala, Uganda; 4 Department of Social Work and Social Administration, College of Humanities and Social Sciences, Makerere University, Kampala, Uganda; 5 Sir Albert Cook Library, College of Health Sciences, Makerere University, Kampala, Uganda; 6 Department of Global Health and Population, Harvard School of Public Health, Harvard University, Cambridge, MA United States of America; 7 Center for Social Science Research on AIDS, Department of Anthropology, College of Arts and Sciences, Case Western Reserve University, Cleveland, OH United States of America; 8 Faculty of Epidemiology and Population Health, London School of Hygiene and Tropical Medicine, London, United Kingdom

**Keywords:** Student, Post-graduate, Research, Knowledge translation, Uganda

## Abstract

**Background:**

Research is a core business of universities globally, and is crucial in the scientific process as a precursor for knowledge uptake and use. We aimed to assess the academic productivity of post-graduate students in a university located in a low-income country.

**Methods:**

This is an observational retrospective documentary analysis using hand searching archives, Google Scholar and PubMed electronic databases. The setting is Makerere University College of Health Sciences, Uganda. Records of post-graduate students (Masters) enrolled from 1996 to 2010, and followed to 2016 for outcomes were analysed. The outcome measures were publications (primary), citations, electronic dissertations found online or conference abstracts (secondary). Descriptive and multivariable logistic regression analyses were performed using Stata 14.1.

**Results:**

We found dissertations of 1172 Masters students over the 20-year period of study. While half (590, 50%) had completed clinical graduate disciplines (surgery, internal medicine, paediatrics, obstetrics and gynaecology), Master of Public Health was the single most popular course, with 393 students (31%). Manuscripts from 209 dissertations (18%; 95% CI, 16–20%) were published and approximately the same proportion was cited (196, 17%; 95% CI, 15–19%). Very few (4%) policy-related documents (technical reports and guidelines) cited these dissertations. Variables that remained statistically significant in the multivariable model were students’ age at enrolment into the Masters programme (adjusted coefficient –0.12; 95% CI, –0.18 to –0.06; *P* < 0.001) and type of research design (adjusted coefficient 0.22; 0.03 to 0.40; *P* = 0.024). Cohort studies were more likely to be published compared to cross-sectional designs (adjusted coefficient 0.78; 95% CI, 0.2 to 1.36; *P* = 0.008).

**Conclusions:**

The productivity and use of post-graduate students’ research conducted at the College of Health Sciences Makerere University is considerably low in terms of peer-reviewed publications and citations in policy-related documents. The need for effective strategies to reverse this ‘waste’ is urgent if the College, decision-makers, funders and the Ugandan public are to enjoy the ‘return on investment’ from post-graduate students research.

The online version of this article (doi:10.1186/s12961-017-0194-8) contains supplementary material, which is available to authorized users.

## Background

Production of research is a core business of universities globally, and is crucial in the scientific process as a precursor for knowledge uptake and use [1]. Consequently, evaluating research outputs would be a proxy for knowledge productivity of health research institutions or groups of scientists [2, 3]. With the considerable investment in research throughout the world, it is imperative to assess the ‘return on investment’ as accountability to funders, research participants, the scientific community as well as the general public [4].

The academic research productivity of post-graduate students can be considered a surrogate measure of universities’ ability to prepare the next generation of health scientists. Imparting research skills is a key aspect of post-graduate training, not only to inculcate scientific inquiry, but also to equip students with the knowledge and skills to critically appraise evidence before applying it [1, 2]. Existing assessments of research productivity by post-graduate students is predominantly from higher income countries [5–7], including a systematic review about residents in the United States of America and Canada or outside sub-Saharan Africa [8–11].

Only two African studies in Cameroon [12] and Zambia [13] documented academic research productivity of post-graduate students in the health sector. Specific to Uganda, existing work on students research has covered either interest and participation of undergraduates in research [14] or mentorship for doctoral candidates [15], but not productivity of their research projects beyond submitting dissertations [16].

The linkage of generation of health research to its use, a process known as knowledge translation, has hardly been studied among post-graduate students worldwide. No studies mapping the pathway of post-graduate students have inquired if these projects go beyond publication of manuscripts from dissertations such as citations in policy-related documents [16].

We aimed to assess the academic research productivity (as measured by dissertations, conference presentations, abstracts and journal articles) and use (as measured by citations) of Makerere University College of Health Sciences (MakCHS), Uganda (previously Makerere Medical School), post-graduate students’ research. We also aimed to explore the determinants of academic research productivity and use.

## Methods

### Study design and justification

Our study was driven by the hypothesis that there is an association between the type of post-graduate degree and the main outcome, which we defined as the proportion of dissertations from which at least one manuscript was published.

We conducted a retrospective cohort documentary analysis and reviewed hard copy records (admissions, research proposals and dissertations) from 1996 to 2010. Further, we performed an electronic search for publications, citations, abstracts and conference presentations in Google Scholar, PubMed and the Makerere University online repository of electronic copies of dissertations from 1996 until June 2016. A systematic review estimated that experimental studies with positive results were published approximately 4 to 5 years after completion, and those with null or negative results in 6 to 8 years [17]. Thus, we considered a range of 6 years (from 2010) to 20 years (from 1996) of study period sufficient to identify publication outputs. We take cognisance of the many meanings and definitions of research productivity [18]. Our choice of outcome variables is informed after review of bibliometric literature, as the more suitable measures of academic research productivity.

### Setting

We conducted this study at MakCHS, formerly Makerere Medical School. MakCHS is the oldest health sciences education and research institution in East Africa, having been established in 1924 [19]. Further, Makerere University is one of the highest ranking post-graduate education and research institutions in Africa [20], providing courses in basic sciences and in clinical and public health disciplines. As such, MakCHS has substantially contributed to the research pool by post-graduate students in Africa.

### Participants and eligibility criteria

Our unit of analysis was an academic research dissertation. We included all Masters level dissertations whose proposals were reviewed by the higher degrees research and ethics committee between 1996 and 2010. These included projects about health-related research topics in the field of veterinary medicine, education, agriculture, or the social sciences or humanities in the spirit of the ‘one-health’ concept to integrate animal, environmental and human health [21], as well as social determinants of health. We excluded research projects conducted by doctorate students or established faculty, except where registered as a Masters student.

### Sampling and sample size

We included all the available 1172 dissertations for Masters students admitted between 1996 and 2010. We opted to study the whole sample since this population was sufficiently small and available.

### Data collection and extraction

We used a data extraction form (Additional file 1) and extracted data from hard copies of research proposals and dissertation reports as well as electronic manuscript publications. We used Google scholar to obtain electronic data about citations and types of documents citing the post-graduate student research. We obtained dissertation records from the directorate of research and graduate training and the office of the academic registrar at Makerere University. We corroborated data on the type and year of post-graduate qualifications with publicly available registers of the national health professional councils (doctors, pharmacists and nurses) who constituted by far the majority of students. We did not corroborate this information for students who qualified with non-medical degrees at undergraduate training, all of whom pursued non-clinical post-graduate degrees, as there is no public registry for them.

### Dependent and independent variables

We measured the primary outcome (dependent variable) for productivity as the proportion of dissertations of research projects by postgraduate students from which at least one manuscript was published in a peer-reviewed journal. Our main secondary outcome was citation of research, measured as the proportion of dissertations by postgraduate students from which the dissertations or manuscripts from dissertations were cited in policy-related documents (technical reports or guidelines) or peer-reviewed journals. As we expected some dissertations to be cited more than once, we considered the first citation by time. Additional outcomes were presentation of abstracts in scientific meetings and uploading into the electronic repository of dissertations at Makerere University Library. We considered publication a key step in the dissemination of research, and thus a precursor for use in decision-making or other applications as appropriate. In addition, we reported the time to any of these outcome events to better understand the average shelf life of student research projects before being translated into tangible outputs or shared for broader usage (publications, conference presentations, citations).

The main independent variable was the type of university degree (biomedical, clinical or public health). We grouped these degrees according to the similarities in the duration of the post-graduate training, nature of the training, time resource available to conduct research as well as size of the programme. As such, Masters of Public Health, Masters of Health Services Research and Masters of Science in Clinical Epidemiology and Biostatistics emphasised research and statistical methods, and were allotted between 2 (full time) or 3 (part time) years to completion. The clinical disciplines (Masters of medicine in surgery, or medicine, or paediatrics, or obstetrics and gynaecology) prioritised practical training in hospital attending to patients, with relatively less time for developing research methodology over a 3-year period of training. Finally, the basic sciences graduate degrees had a substantial component of laboratory-based work.

Additional independent variables were demographics of post-graduate students (age, sex, marital status), research environment (funding, period), research design (quantitative or qualitative), the level of research (sub-individual, individual and population) and priorities (Millennium Development Goals (MDGs) or the health system strengthening building blocks of WHO) [22, 23]. This description of levels of research pertains to sub-individual (laboratory), individual (clinical) and population (public health) levels [24].

### Categorising the data

In terms of health priorities, we categorised studies according to the health-related MDGs [22], with MDG 1 on ending hunger; MDG 4 on child health; MDG 5 on maternal health; MDG 6 on HIV/AIDS, malaria, TB and other diseases; and MDG 7 on environmental health (water and sanitation). We used the framework of the WHO pillars for health system strengthening to assess the post-graduate research projects (governance, financing, human resources, service delivery, information systems and access to medicines, vaccines health technologies) [23].

Secondly, we organised the student projects into the three periods that may have influenced the research and health policy environment. The first period was from 1996 to 2000, after a call for research investment considering the 10/90 gap [25]. In brief, this highlights inequity in research investment (10%), where the bulk (90%) of health problems are commonly in low-income countries. The second period was from 2001 to 2005, at the start of MDGs [22], while the third was from 2006 to 2010, which would depict more recent events such as the 2004 and 2008 inter-ministerial summits in Mexico and Mali, respectively, about the use of research in health decision-making and policy [26]. Noteworthy, the Makerere Medical School transitioned into the collegiate system in 2007, the third period that could have attracted or altered the distribution of human and other resources, as well as prioritisation of research at the school and department levels. Our final classification addressed the types of research design (quantitative or qualitative) and research level as described under the variables section [24].

### Data analysis

We analysed the data using Stata version 14.1 software and relevant health research priorities frameworks for WHO and the MDGs. We used frequencies, proportions and measures of central tendency to conduct initial analyses and tabulated these. We used multivariable logistic regression to explore the determinants of productivity (publication) and use (citations) of student’s research. In the multivariable logistic regression model we included covariates that altered the relationship between the primary outcome (publication) and main exposure (degree type) by more than 10%. We chose these covariates for testing a priori guided by literature but also our expert knowledge in the field of health systems and health policy. In the time-to-event analysis, we analysed for events after completion of dissertation project. We used two sided tests with *P* < 0.05 as the significance level.

## Results

### Description of post-graduate students and research projects

We found dissertations of 1172 Masters students. We excluded 51 doctoral dissertations, whereas no Masters level dissertation met the exclusion criteria. These dissertations were written by students of predominantly male sex (69%), of whom nearly half were married (49%) and with a mean age of 32 years (SD, 5.2) at the time of enrolment into their post-graduate course. While half (590, 50%) of them had completed clinical graduate disciplines (surgery, internal medicine, paediatrics, obstetrics and gynaecology), Master of Public Health was the single most popular course, with 363 students (31%). Half of students (49%) had scholarship funding (Table 1).

**Table 1:** Characteristics of post-graduate students at Makerere University College of Health Sciences, 1996–2010, *N* = 1172

Characteristic	N (%)
Age (mean, SD)	32 (5.2)^a^
Sex (female)	360 (31)
Married	569 (49)
Basic sciences Master’s degree^d^	127 (10)
Clinical Master’s degree	
MMed Paediatrics	118 (10)
MMed Internal medicine^b^	155 (13)
MMed Surgery^c^	200 (17)
MMed Obstetrics and gynaecology	117 (10)
Public health/research degree	
MPH	363 (31)
MHSR	16 (1)
MSc EpiBio	76 (6)
Period of registration	
1996–2000	246 (21)
2001–2005	475 (41)
2006–2010	451 (38)
Sponsorship	
Funded (Agency)	570 (49)
Self-funded or not reported	602 (51)

^a^All values are frequencies and proportions are in parentheses, except for the variable ‘Age’, where the mean and standard deviation are depicted

^b^Includes MMed Psychiatry and MMed Family Medicine

^c^Includes dentistry and surgical sub-specialties (ophthalmology, ear nose and throat)

^d^MMed/MSc basic sciences includes anatomy, anaesthesiology, clinical psychology, microbiology, pathology, pharmacology, physiology and radiology; these have been grouped together due to their low admission numbers

*MMed* Masters of Medicine, *MPH* Masters of Public Health, *MHSR* Masters Health Services Research, *MSc* Master of Science, *EpiBio* Clinical Epidemiology & Biostatistics

In terms of research priorities (Table 2), the bulk of the research was about MDG 6 predominantly on infectious diseases (42%) and non-communicable diseases (33%), whilst social determinants of health in MDG 1 on nutrition and hunger (4%) and MDG 7 on environmental health (2%) were the least researched. The health services delivery pillar (66%) was the most common health system pillar researched, with few studies on governance (1%), financing (1%) and human resources for health (2%).

**Table 2:** Types of research projects by priority areas and study design at Makerere University College of Health Sciences

Priority areas	*N* = 1172
MDGs	
Child health	211 (18)
Maternal health	184 (16)
Infectious diseases	493 (42)
HIV	246 (21)
TB	71 (6)
Malaria	55 (5)
NTDs	16 (1)
Other IDs	105 (9)
NCDs	380 (33)
CVD	51 (4)
DM	24 (2)
Mental illness	68 (6)
Cancer	68 (6)
Injury	75 (6)
Other NCDs	96 (8)
Nutrition/hunger	50 (4)
Water/sanitation	23 (2)
WHO HSS pillar	
Services delivery	776 (66)
Human resources	20 (2)
Supply chain	11 (1)
Information systems	11 (1)
Governance	10 (1)
Financing	9 (1)
Study design	*N* = 1172
Quantitative design	
Cross-sectional	879 (75)
Cohort	95 (8)
Case control	74 (7)
Randomised trials	58 (5)
Diagnostic accuracy	39 (3)
Economic evaluation	12 (1)
Qualitative design	
Case study	241 (21)
Narratives	4 (0.3)
* Data collection* ^a^	
Focus group discussions	170 (15)
Key informant interviews	85 (7)
In-depth interviews	6 (0.3)
Level of research^b^	
Clinical	803 (69)
Public health	329 (28)
Services	189 (16)
Epidemiology	111 (10)
Policy	15 (1)
Resources	14 (1)
Laboratory	28 (2)

^a^Distinguishes qualitative data collection methods from qualitative study designs per se

^b^Description of research at sub-individual (laboratory), individual (clinical) and population (public health) levels

*HSS* health systems strengthening, *MDG* Millennium Development Goals, *WHO* World Health Organization, *CVD* cardiovascular disease, *DM* diabetes mellitus, *HIV* human immunodeficiency virus, *IDs* infectious diseases, *NCDs* non communicable diseases, *NTDs* neglected tropical diseases, *TB* tuberculosis

Three quarters (75%) of the post-graduate research projects were cross-sectional studies, with the least common designs being randomised trials (5%), diagnostic accuracy (3%) and economic evaluation (1%). A fifth (21%) used qualitative case-study methodology, with focus discussion groups (15%) being the most popular qualitative data collection method. We distinguished qualitative study designs from data collection methods, underscored in Table 2.

The post-graduate student research was mainly at the individual (69%) and population levels (28%), with only 28 studies at sub-individual level (Table 2). There was a high correlation between the level of research conducted and the Masters degree discipline. We found 70% of the individual level research work was done in the clinical disciplines (surgery, internal medicine, paediatrics, obstetrics and gynaecology), 93% of population level inquiries were in public health or research Masters degree courses, while 82% of the sub-individual level studies were conducted within basic sciences disciplines (data not tabulated).

### Outcomes of research projects by postgraduate students

Over the 20-year period of the study, manuscripts from 209 dissertations (18%; 95% CI, 16–20%) were published in peer-reviewed journals without any difference over the three time periods. The earliest time to publication was 12 days after submission of the dissertations, while the longest was 12 years (data not tabulated). The median time to publication from completion of the dissertation write up was 2.3 years (interquartile range (IQR), 1.4–3.7). There was a time reduction in the third period to 2 years from the first two periods from 2.5 and 2.8 years, respectively, and this was statistically significant (*P* < 0.001) (Table 3).

**Table 3:** Research project outcomes by post-graduate students at Makerere University College of Health Sciences, 1996–2010

Outcome	*N* (%; 95% CI)	Period and number of registered students (%)		
		1996–2000	2001–2005	2006–2010
		246 (21%)	475 (41%)	451 (38%)
Primary				
Journal article	209 (18%; 16–20%)	41 (17%)	78 (16%)	86 (19%)
Secondary				
Citation	196 (17%; 15–19%)	42 (16%)	82 (17%)	72 (17%)
Conference presentation	21 (2%; 1–3%)	4 (2%)	9 (2%)	8 (2%)
Dissertation (electronic)	465 (40%; 37–43%)	56 (23%)	191 (40%)	218 (48%)
Combined				
≥ One outcome	582 (50%; 47–52%)	89 (36%)	240 (50%)	252 (56%)

We report that approximately the same proportion of dissertations were cited (17%, 95% CI, 15–19%) as to those that were published. Additionally, there was a high and significant correlation between publication and citation (*r* = 0.8, *P* < 0.0001). However, on further analysis, not all publications were cited (*n* = 38, 18%) and not all citations were from peer-reviewed publications (*n* = 25, 13%). The median time to first citation after completion of research projects was 3.8 years (IQR, 2.6–5.5). The median time to first citation increased from 4 years in the first period (IQR, 3.1–6.1) to 5.2 years in the second (IQR, 2.9–6.8), before significantly declining to 2.7 years in the third (IQR, 1.9–3.8; *P* < 0.001) (Table 4).

**Table 4:** Time to research event by post-graduate students at Makerere University College of Health Sciences, *N* = 1172

Outcome	Years, median (IQR)	Period		
		1996–2000	2001–2005	2006–2010
Primary				
Journal articles	2.3 (1.4–3.7)	2.5 (1.5–4.2)	2.8 (1.7–4.4)	2 (1–2.5)
Secondary				
Citation	3.8 (2.6–5.5)	4 (3.1–6.1)	5.2 (2.9–6.8)	2.7 (1.9–3.8)
Conference presentation	0.7 (0.3–1)	0.9 (0.6–5.7)	1 (0.3–1)	0.6 (–0.7 to 0.8)
Thesis report (book)	2.7 (2.3–3)	2.6 (1.9–2.7)	2.7 (2.5–3.3)	2.7 (2.4–3.1)
Dissertation (final book)	3 (2.8–3.8)	2.9 (2.6–3.1)	3 (2.7–3.8)	3.1 (2.9–4)
Dissertation (electronic)	3.8 (2.8–7.2)	10.3 (10.5–11.5)	4.4 (3.4–7.2)	2.7 (1.9–3.4)

Most of the first-citing documents (81%) were peer-reviewed journals, followed by dissertations (15%; Masters (*n* = 15) and Doctoral (*n* = 13)) and very few (4%) policy-related documents (technical reports and guidelines). These policy-related documents were from WHO (*n* = 3) and one each for the Uganda Red Cross Society, Bill and Melinda Gates Foundation, United States Agency for International Development, and Medscape® online guidelines (http://www.medscape.com/). Among the peer reviewed cited documents only one was a systematic review and only two were book chapters (data not tabulated).

Two of five (40%) of the hard copy dissertations were accessible in the online repository of electronic dissertations at MakCHS. There was a substantial and significant increase over time, from 23% (95% CI, 13–23%) in period one (1996–2000) to 40% (95% CI, 36–45%) and 48% (95% CI, 44–53%) in the next two periods, respectively (*P* < 0.001). The first dissertation was uploaded electronically in November 2011, when the online institutional repository was commissioned by Makerere University. The median time to electronic repository of dissertations was 3.8 years (IQR, 2.8–7.2). This varied from 10.5 years in period 1 (IQR, 10.3–11.5) to 4.4 years (IQR, 3.4–7.2) and 2.7 years (IQR, 1.9–3.4) in periods 2 and 3; these changes were statistically significant (*P* < 0.001).

Finally, we found that only 2% of the dissertations were presented in scientific conferences, all of which were outside Uganda. The median time to presentation of abstracts in scientific meetings from completion of research projects was 0.7 years (IQR, 0.3–1). There was an absolute reduction from 0.9 years and 1 year in the first two periods to 0.6 years in the third, but this was not statistically significant (*P* = 0.241).

### Determinants of publication of research by postgraduate students

The unadjusted regression model suggested that research performed by clinical post-graduate students was more likely to be published (adjusted coefficient, 0.29; 95% CI, 0.07–0.52; *P* = 0.01) when compared to the public health or research disciplines (health services research or clinical epidemiology and biostatistics or public health). However, the variables that remained statistically significant in the multivariable model were students age at enrolment into the Masters programme (adjusted coefficient, –0.12; 95% CI, –0.18 to –0.06; *P* < 0.001) and type of research design (adjusted coefficient, 0.22; 0.03–0.40; *P* = 0.024). Cohort studies were more likely to be published compared to cross-sectional designs (adjusted coefficient, 0.78; 95% CI, 0.2–1.36; *P* = 0.008). The ‘level of research’ had borderline significance by *P* = 0.098, with an adjusted coefficient of 0.78 (95% CI, –0.14 to 1.71), although the confidence interval included zero, as shown in Table 5. The level of research was driven by sub-individual (laboratory) studies (adjusted coefficient, 2.36; 95% CI, 0.06–4.65; *P* = 0.044). The main independent variable (graduate degree type), sex, marital status and scholarship funding were not statistically significant at all in the multivariable model.

**Table 5:** Determinants of peer reviewed publication of post-graduate research at Makerere University College of Health Sciences

Determinant	Multivariable		
	Adjusted coefficient^a^	95% CI	*P* value
Degree type/Department	–0.22	–0.65 to 0.21	0.313
Age	–0.12	–0.18 to –0.06	<0.001
Married	0.32	–0.15 to 0.79	0.177
Funding support	0.12	–0.35 to 0.59	0.612
Research priority WHO	–0.05	–1.37 to 1.26	0.937
Research design	0.22	0.03 to 0.40	0.024
Research level	0.78	–0.14 to 1.71	0.098
Constant	1.36	–0.61 to 3.33	0.176

^a^Adjusted coefficient – the regression coefficient adjusted for all other confounding variables in the model

*CI* confidence interval

## Discussion

### The principal findings

We report low publication productivity among post-graduate students at Makerere University. In addition, older students published less and cohort studies were more likely to be published. In terms of first citations, one in five dissertations were cited mostly in peer-reviewed journal articles. At least one in 20 dissertations were cited in policy-related documents, signifying use in the policy process mostly by international actors.

### Findings in relation to other studies

Most of the research work by post-graduate students at Makerere University remains unpublished and therefore less accessible to end-users including decision makers. Our finding of a low publication proportion (18%) is corroborated by studies in Cameroon [12], Egypt [9] and India [8] at 14%, 20% and 30%, respectively. Similar studies in higher income countries reported higher but still sub-optimal publication at 40%, 45%, 45%, 65% and 66% in Iran [27], New Zealand [5], Canada [28], Spain [29] and the United States [30], respectively.

Publication of post-graduate research findings is necessary, but not sufficient for its utility. Our paradoxical finding in publication versus citation of post-graduate research is substantiated in recent paper, that about 31% of published World Bank reports were never read (not downloaded at all) [31]. Therefore, publication is not a guarantee that these results will be immediately useful. Nonetheless, publishing, particularly in open access journals, will, at the very least, nourish the body of empirical evidence readily available for use.

Post-graduate students’ dissertations were barely used to inform policy or practice guidelines. That most citations were by peer-reviewed articles suggests a predominant use in informing further research, with far less application in policy-related processes. What, then, could explain this very low utility? In two systematic reviews, decision-makers perceived publications aimed at a scholarly audience and irrelevance of publications as two impediments to the uptake of research for decision-making [32, 33]. Clearly, the perennial health system challenges in low-income countries (health financing, governance and human resources) were under-researched, lending currency to the argument of irrelevance of the post-graduate students research. However, our results show alignment with health-related MDG priorities and Uganda’s disease burden, as further corroborated by a study of the University of Zambia [13]. Perhaps this is an artefact of what is emphasised by academia (peer-reviewed publications versus evidence briefs for policy) or a documentation deficiency (including poor indexing of policy-related documents) or a selection bias, since most of these policy-related documents were of international agencies that we retrieved online. Until recently, Ugandan and Cameroonian policy documents rarely included references to research, reflecting the same trend in low-income settings [34]. Assessing how research is used remains a subjective process without documented hard evidence, a subject for further inquiry.

Younger age at enrolment into the post-graduate courses independently predicted publication in peer-reviewed journals; the fact that publication output reduced among older post-graduate students may reflect shifting priorities or social responsibilities and resultant opportunity costs for the limited time [5]. Noteworthy, marital status, which would imply less time available for research, did not impact on productivity of publications in the multivariable model in our study. Regardless of this finding, newly qualified post-graduate students are commonly caught up finding a job or career openings without time to think of immediate publication. This is a plausible explanation given that it took at least 2 years after dissertation completion to publish approximately 1 in 40 manuscripts from these dissertations. Our findings are augmented by studies from similar settings. Among the Doctoral and Masters dissertations in Egypt that were published, 63% were published after the first year [9]. In an Indian study, only 8% of the dissertations were published in the first year after completion of the Masters degree [8].

Since the type of research design used was a determinant of publication irrespective of scholarship funding, it is tempting to suggest that the quality of evidence from the research influenced publication. However, this contradicts a recent study where cross-sectional designs were significantly published more by pharmacy residents in the United States [35]. Dhaliwal et al. [8] showed that study design did not influence publication in India. Probably, peer-reviewer bias could have had an important bearing on the types of studies by post-graduates at Makerere University that eventually got published. It is plausible that such post-graduate students underwent their dissertation work using already established cohorts of patients that were managed by large collaborative programs. One could argue that they were more likely to be published because of the push from the partners managing the projects that supported the research.

The finding that only 2% of abstracts were presented in conferences may be misconstrued as low-level dissemination efforts. However, international conferences abroad are likely to have put their conference abstracts or proceedings online, whilst those organised in Uganda or within the African region preferred hard copy abstracts and reports without online archives.

### Strengths and limitations

We highlight several strengths and a few limitations in our review. The strengths lie in the new information on previously un-researched outcomes (citation of research) and time-to-event indicators, among post-graduate students. The breadth of health disciplines covered (biomedical, clinical and public health), a large sample size and a long study period make our findings even more robust. Nonetheless, we encountered some shortcomings, particularly with regards to missing data or inaccuracies due to limited availability of records dating 20 years back. Although we used multiple corroborating sources to fill in information gaps, we did not perform multiple imputations of the missing data in the regression models. We acknowledge that citation of research is one of the many ways to identify research use. Still, it was challenging to document the outcome of use in policy-related documents, which we likely underestimated. Despite our anticipation of fewer outcomes in the third period towards 2010 because of a shorter study time period, this was not the case. Interestingly, what we observed was a general reduction in the time to outcomes (publication, citation and conference presentations), which could be explained by bias in the longer continuation of the first two periods or increased advocacy, supervision and funding support to publish post-graduate students research. We did not assess local use of the results of post-graduate research dissertations, say in the field at the level of district, by district health teams (as would be the case for Masters of Public Health students) or management of patients (as would be the case for clinical disciplines). Similarly, we may have missed abstract presentations in local conferences that do not have electronic records posted online. It is possible that publication bias, which is the tendency for investigators to submit manuscripts and of editors and reviewers to accept them, based on the strength and direction of the research findings, may account for the low publication proportion [36]. We thus urge readers to interpret our results within this context.

### Implications for policy on post-graduate research

Our results have implications for stakeholders in the post-graduate training enterprise at national and global level. First, we have documented the deficiency in publishing post-graduate research dissertations in a leading education and research training institution in Africa. Secondly, we have provided empirical evidence that post-graduate research is cited in policy-related documents, however minimal. Considered together, this calls for investment in promoting effective interventions that increase publication [37] and active knowledge translation approaches that will link this research to policy [32, 33]. A low-cost intervention such as obligatory publication of Masters dissertations may positively impact on productivity, particularly by scholarship funders, as is the case for doctoral students at Makerere University. Whichever intervention is chosen should consider the unique circumstances of older post-graduate students. Last but not least, education research funders should prioritise impact evaluation studies on post-graduate research dissertations.

### Implications for future research

We identified opportunities for further inquiry. Most studies have emphasised productivity and citations, yet the ultimate goal is for this research to inform decision-making or policy formulation and hopefully improve population health. Research may be used conceptually (to enlighten the decision maker), instrumentally (to solve a problem at hand), or symbolically (political or tactical use to justify action or inaction) [38, 39]. Although it is likely that post-graduate research was used either conceptually or instrumentally we did not distinguish the two. An in-depth qualitative investigation would inform how post-graduate research is used as well as identify potential interventions for knowledge translation in the local Ugandan policy environment.

Indeed, the ground is fertile for quasi-experimental designs testing interventions that have shown some promise in raising productivity elsewhere or prospective cohort studies assessing broader outcomes such as number of dissertations submitted for publication and those rejected, or comparisons with doctoral students or established faculty. Finally, mixed methods analyses about where and why post-graduate students publish (or not), including citometrics (impact factor), which were beyond the scope of our study, would improve understanding about the context-specific interventions to increase research productivity.

## Conclusions

The academic productivity and citations of post-graduate students research conducted at MakCHS is considerably low in terms of peer-reviewed publications and citations in policy-related documents, respectively. The need for effective strategies to reverse this ‘waste’ is urgent if Makerere University, decision makers, funders and the Ugandan public are to enjoy the ‘return on investment’ from this knowledge mine of post-graduate students research.

## Additional file

Additional file 1: Data Collection Tool For The Study Of Knowledge Translation Of Post-Graduate Students Research In Uganda (KTPG-Study). (PDF 28.2 kb)
