# Supplementary material for: Academic research productivity of post-graduate students at Makerere University College of Health Sciences, Uganda, from 1996 to 2010: a retrospective review
Source: Health Res Policy Syst. 2017 Apr 4;15:30. doi: 10.1186/s12961-017-0194-8 (PMC5381020; doi:10.1186/s12961-017-0194-8)
Supplement: Additional file 1: — Data Collection Tool For The Study Of Knowledge Translation Of Post-Graduate Students Research In Uganda (KTPG-Study). (PDF 28.2 kb) [file 12961_2017_194_MOESM1_ESM.docx]

**DATA COLLECTION TOOL FOR THE STUDY OF KNOWLEDGE TRANSLATION OF POST-GRADUATE STUDENTS RESEARCH IN UGANDA (KTPG-Study)**

|  |  |  |
| --- | --- | --- |

1. **Unique Identification Numb. (Reg. No.):**

|  |  |  |
| --- | --- | --- |

1. **Initials of data officer:**

| **Day** | **Month** | **Year** |
| --- | --- | --- |
|  |  | **2015** |

1. **Date:**

| **8 am–12pm** | **12 – 5pm** | **5 – 7pm** |
| --- | --- | --- |
|  |  |  |

1. **Time:**

**PART A: STUDENT ADMINISTRATIVE INFORMATION**

1. Name: ___________________________________ DOB: ____________________ Gender: ______
2. Tel.: ___________________ Email address: ________________________Marital status: ________
3. Supervisor 1: ______________________________Supervisor 2: ___________________________
4. Sponsorship (name): ⬜ Self or ⬜ Funder______________________________________________

**PART B: POST – GRADUATE RESEARCH PROJECT**

1. Date completed **undergraduate** **degree** (see transcript): __________________________________
2. Undergraduate degree **course** (see transcript): __________________________________________
3. Date of post-graduate **admission** (see admission letter): ___________________________________
4. Date of thesis **submission** (see letter of student): ________________________________________
5. Date of **viva voce** (see pre–award report): ______________________________________________
6. Date of thesis **completion** (see report of thesis corrections reviewer): ________________________
7. Date of post graduate **award** (see letter of award): _______________________________________
8. Post graduate **school** and department/unit: _____________________________________________
9. Post graduate course (Full Time/Distance Education): ____________________________________
10. Thesis title/main objective: _________________________________________________________

______________________________________________________________________________________________________________________________________________________________________

**PART C: RESEARCH QUESTION CLASSIFICATION**

1. Type of research by the Julio Frenk classification *(tick all that apply)*:

⬜ Clinical

⬜ Laboratory based/Biomedical

⬜ Public Health – health systems – policy

⬜ Public Health – health systems – organization – services

⬜ Public Health – health systems – organization – resources

⬜ Public Health – epidemiology – determinants

⬜ Public Health – epidemiology – consequences

⬜ Not Applicable/Not reasonably categorized by MDG

1. Type of research by epidemiological (and qualitative) study design *(tick all that apply)*:

⬜ Quantitative ⬜ RCT ⬜ Cohort ⬜ Case–control ⬜ Cross–sectional

⬜ Qualitative ⬜ Case study ⬜ Grounded theory ⬜ Phenomenology ⬜ Historical

⬜ Mixed Methods (Quantitative and Qualitative) ⬜ FGDs ⬜ KII ⬜ In-depth Interviews

⬜ Other (specify) ___________________________________________________________

1. Type of research by Millennium Development Goals *(tick all that apply)*:

⬜ MDG 1 – Nutritional Health

⬜ MDG 4 – Child Health

⬜ MDG 5 – Maternal Health

⬜ MDG 6 – IDs: ⬜ HIV/AIDS ⬜ Malaria ⬜ TB ⬜ NTD ⬜ Other_________________

⬜ MDG 6 – NCDs: ⬜ Mental Health ⬜ CVD ⬜ DM ⬜ Liver ⬜ Kidney ⬜ Other_____

⬜ MDG 7 – Environment: climate change, water and sanitation (e.g. microbiological contamination)

⬜ Not Applicable/Not reasonably categorized by MDG

1. Type of research by World Health Organization pillars of Health Systems Strengthening *(tick all that apply)*:

⬜ Leadership, governance and management

⬜ Health financing arrangements

⬜ Health workforce (human resources for health)

⬜ Health services delivery

⬜ Health information systems

⬜ Access to medical technologies, medicines and vaccines

⬜ Not Applicable/Not reasonably categorized by Health Systems Strengthening
